# The global prevalence of headache disorders of public-health importance: a meta-analysis of population-based individual participant data from 41,614 adults from 17 countries

**DOI:** 10.1186/s10194-025-02142-9

**Published:** 2025-10-07

**Authors:** Andreas Kattem Husøy, Shengyuan Yu, Ruozhuo Liu, Akbar A. Herekar, Bilal Ahmed, Arif D. Herekar, Callixte Kuate Tegueu, Anastase Dzudie Tamdja, Annick Mélanie Magnerou, Najib Kissani, Latifa Adarmouch, Thierry Adoukonou, Mendinatou Agbetou, Oyéné Kossi, Mehila Zebenigus, Redda Teckle-Haimanot, Dawit K. Worku, Girish N. Rao, Girish B. Kulkarni, Gopalkrishna Gururaj, Mohammed Al Jumah, Ali M. Al Khathaami, Guiovanna Quispe, Carlos Palomino-Diaz, Mattias Linde, Ajay Risal, Krishnan Anand, Debashish Chowdhury, Ashish Duggal, Otgonbayar Luvsannorov, Selenge Enkhtuya, Dorjkhand Baldorj, Ilya Ayzenberg, Zaza Katsarava, Colette Andrée, Gretchen L. Birbeck, Elena Ruiz De la Torre, Daiva Rastenytė, Hallie Thomas, Lars Jacob Stovner, Timothy J. Steiner

**Affiliations:** 1 Department of Neuromedicine and Movement Science, NorHead, NTNU, Norwegian University of Science and Technology, Edvard Griegs gate, Trondheim, Norway; 2 Department of Neurology and Clinical Neurophysiology, St Olavs University Hospital, Trondheim, Norway; 3 Department of neurology, Chinese PLA General Hospital, Beijing, China; 4 Headache Research Foundation of Pakistan, Karachi, Pakistan; 5 Department of Anesthesiology and Perioperative Medicine, Medical College of Georgia, Augusta, GA USA; 6 Department of Emergency Medicine, Manchester Royal Infirmary, Manchester University Foundation Trust, Manchester, UK; 7 Department of Neurosciences, Baqai Medical University, Karachi, Pakistan; 8 Department of Neurology, Douala Laquintinie Hospital, Douala, Cameroon; 9 Faculty of Medicine and Biomedical Sciences, University of Yaoundé, Yaoundé, Cameroon; 10 Department of Internal Medicine, Douala General Hospital, Douala, Cameroon; 11 Clinical Research Education, Networking and Consultancy (CRENC), Yaoundé, Cameroon; 12 Laboratory of Clinical and Experimental Neuroscience, Faculty of Medicine, Cadi Ayyad University, Marrakech, Morocco; 13 Department of Neurology, Mohammed VI University Hospital, Marrakech, Morocco; 14 Community Medicine and Public Health Department, Bioscience and Health Research Laboratory, Faculty of Medicine, Cadi Ayyad University, Marrakech, Morocco; 15 Department of Neurology, University of Parakou, Parakou, Benin; 16 Department of Neurology, School of Medicine, College of Health Science, Addis Ababa University, Addis Ababa, Ethiopia; 17 Department of Internal Medicine, School of Medicine, College of Health Science, Addis Ababa University, Addis Ababa, Ethiopia; 18 Department of Internal Medicine, Bahir Dar University, Bahir Dar, Ethiopia; 19 Department of Epidemiology, National Institute of Mental Health and Neuro Sciences (NIMHANS), Bangalore, India; 20 Department of Neurology, National Institute of Mental Health and Neuro Sciences (NIMHANS), Bangalore, India; 21 King Abdullah International Medical Research Centre, Riyadh, Saudi Arabia; 22 King Saud Bin Abdulaziz University for Health Sciences, Riyadh, Saudi Arabia; 23 InterHealth Hospital, Riyadh, Saudi Arabia; 24 King Abdulaziz Medical City, Riyadh, Saudi Arabia; 25 Neurology Service, Hospital Luis Negreiros Vega, Callao, Peru; 26 Regional Migraine Unit, Sahlgrenska University Hospital, Gothenburg, Sweden; 27 Department of Psychiatry, Kathmandu University School of Medical Science, Dhulikhel, Nepal; 28 All India Institute of Medical Sciences, New Delhi, India; 29 GB Pant Institute of Postgraduate Medical Education and Research, New Delhi, India; 30 Department of Neurology, Mongolian National University of Medical Sciences, Ulaanbaatar, Mongolia; 31 Department of Neurology, St. Josef-Hospital, Ruhr-University Bochum, Bochum, Germany; 32 Centre of Neurology, Geriatric Medicine and Early Rehabilitation, Evangelical Hospital, Unna, Germany; 33 Medical Faculty, University of Essen, Essen, Germany; 34 Center of Public Health Research, CRP-Santé, Strassen, Luxembourg; 35 Department of Pharmaceutical Sciences, University of Basel, Basel, Switzerland; 36 Neurology Research Office, University Teaching Hospitals, Lusaka, Zambia; 37 Department of Neurology, University of Rochester, Rochester, New York US; 38 European Migraine and Headache Alliance, Brussels, Belgium; 39 Lithuanian University of Health Sciences, Kaunas, Lithuania; 40 Department of Neurology, University of Copenhagen, Copenhagen, Denmark; 41 Division of Brain Sciences, Imperial College London, London, UK

**Keywords:** Migraine, Tension-type headache, Medication-overuse headache, Prevalence, Population-based study, Individual participant data, Meta-analysis, Global burden of disease (GBD), Global campaign against headache

## Abstract

**Background:**

Recent studies indicate that migraine affects 14–15% of the global population, tension-type headache (TTH) around 26%, and medication-overuse headache (MOH) 1–2%. While these estimates highlight the impact of these conditions on population health, their reliability is compromised by the variable quality of contributing studies. In response, the Global Campaign against Headache has supported epidemiological studies in all parts of the world, using standardized methods.

**Methods:**

We conducted a meta-analysis of individual participant data from these studies, accepting only population-representative data (17 countries from all world regions). All included studies were cross-sectional surveys of adults aged 18–65 years using the Headache-Attributed Restriction, Disability, Social Handicap and Impaired Participation (HARDSHIP) questionnaire. Algorithmic diagnosis applying modified International Classification of Headache Disorders (ICHD) criteria identified the headache disorders of public-health importance: migraine, TTH and probable MOH (pMOH: the association of headache on ≥ 15 days/month [H15+] and reported medication overuse). Two sets of estimates were made for migraine and TTH, one excluding those with H15+ (standard process), the other including these (extended process). We analysed associations with demographical variables, and, accordingly, adjusted prevalence estimates for age, gender and country income level.

**Results:**

We included 41,614 individuals, with over-representations of females (22,278 [53.5%]) and of participants from lower-middle income countries (59.7%; global 37.8%). Age-distribution was similar to that of the world. Overall, 65.5% (95% CI: 65.0–66.0) reported headache during the previous year, females (72.1% [71.5–72.7]) more than males (57.9% [57.2–58.6]). Migraine was more common among females (standard process: 29.5% [28.9–30.1]; extended process: 33.1% [32.5–33.7]) than males (18.6% [18.1–19.2]; 20.1% [19.6–20.7]), as was pMOH (5.6% [5.3–5.9] vs. 2.3% [2.1–2.5]). TTH was similarly prevalent among males (33.4% [32.8–34.1]; 34.9% [34.2–35.5]) and females (31.2% [30.6–31.8]; 33.2% [32.6–33.8]). Headache was more prevalent in high/upper-middle income countries (71.4% [70.6–72.2]) than in low/lower-middle income (63.0% [62.5–63.6]). Prevalence estimates adjusted for age, gender and income level were 65.0% (64.6–65.5) for any headache, 23.5% (23.1–23.9; standard process) and 25.9% (25.4–26.3; extended process) for migraine, 33.2% (32.7–33.6) and 34.7% (34.3–35.2) for TTH, and 4.1% (3.9–4.3) for pMOH.

**Conclusions:**

About 65% of the world’s population aged 18–65, likely to include most of the world’s workforce, will have headache during the coming year, about 25% migraine and 4% pMOH. Both these estimates are higher than previous comparable estimates, and we believe them to be the best currently available for this population. Our estimate of TTH prevalence (about 33%) is probably substantially too low, since methodological constraints precluded identification of TTH in those with concomitant migraine.

**Supplementary Information:**

The online version contains supplementary material available at 10.1186/s10194-025-02142-9.

## Background

Recent meta-analyses and reviews of the literature have found the global 1-year prevalence of migraine to be 14–15% [1–5], of tension-type headache (TTH) around 26% [2–5] and of medication-overuse headache (MOH) 1–2% [6–9]. These are the three headache disorders of public-health importance. However, many of the data contributing to these estimates are of questionable reliability, being derived from studies of variable quality and not all of sound methodology [10].

Recognising this, the Global Campaign against Headache has, over the last two decades, fostered and supported the conduct of studies estimating not only the prevalence but also the attributable burden of headache disorders among adults (aged 18–65 years) from countries in all parts of the world [11, 12]. The programme has been led by *Lifting The Burden* (LTB), a UK-registered charitable non-governmental organization, but the enormous workload – collecting data from more than 47,000 participants in 28 samples from 24 countries – has been shared by all authors. With comparability being the driving objective, the studies have used standardized consensus-based methodology [10, 13], albeit with some necessitated departures (see Methods), and the same questionnaire (the Headache-Attributed Restriction, Disability, Social Handicap and Impaired Participation [HARDSHIP] questionnaire [4]). Most have used random cluster-based sampling to obtain representative population samples, and face-to-face interviews conducted during unannounced household visits.

The published findings from these studies provide robust evidence that the prevalence of migraine (the simple mean being 22.6%), if not those of TTH and MOH, is substantially underestimated. It should be noted, however, that these data are derived exclusively from adults aged 18–65 years (headache prevalence may be lower among both children and the elderly), and included probable migraine and probable TTH, an issue discussed later.

The individual participant data (IPD) from all of these studies have been pooled into the HARDSHIP adult database [12], hosted by the Norwegian Centre for Headache Research (NorHEAD) at the Norwegian University of Science and Technology (NTNU). These IPD enable powerful meta-analyses, with the use of IPD considered methodologically superior to pooling aggregate data from different studies [14].

Our aims were two-fold: first, to provide strongly evidence-based global prevalence estimates for these three headache disorders among adults aged 18–65 years (likely to include most of the world’s workforce), and, second, to investigate associations between the prevalence of each and demographic variables, notably age, gender and country income level.

## Methods

### Ethics

All contributing studies were approved by the appropriate local ethics committees, and conducted in accordance with the principles of the Declaration of Helsinki [15] (details are reported in the respective publications [16–31]).

All participants gave consent (either verbal or written, in accordance with local requirements) to their participation in the original studies.

All data were anonymous.

### Study design and data acquisition

Supplementary Table 1 details the characteristics of all studies (28 samples from 24 countries) in the HARDSHIP adult database [12].

We included the IPD from 18 of the samples: from Benin [16], Cameroon [17, 32], China [18, 33], Ethiopia [19, 34], India (Karnataka State [20, 35] and Delhi and North Capital Region [NCR] [31]), Lithuania [21, 36], Luxembourg [36], Mongolia [23, 37], Morocco [24, 38], Nepal [25, 39], Netherlands [36], Pakistan [40], Peru [27, 41], Russia [28], Saudi Arabia [29, 42], Spain [36] and Zambia [30, 43]. All contributing studies used standardized methodology for sampling, engagement with participants and enquiry [10, 13], but with local adaptations necessary in some, which are detailed below. All were cross-sectional surveys of adults aged 18–65 years, recruiting participants through cluster-based random sampling. In most studies, during unannounced visits at randomly selected households within each cluster, one eligible adult, also randomly selected, was interviewed face-to-face. However, the studies in Luxembourg [36], Netherlands [36], Spain [36] and Saudi Arabia [29] deviated from this procedure. Participants from Luxembourg and Netherlands came from stratified samples of the general population contacted by regular post and internet respectively [36]. Participants from Spain were postal service employees contacted through the employer’s internal post [36]. In Saudi Arabia, culture precluded unannounced household visits, and participants were contacted by cell-phone through random digit dialling [29].

We excluded 10 samples (Supplementary Table 1). The sample from Mali lacked diagnostic information necessary to diagnose migraine and TTH [44]. Nine other samples had been vulnerable to bias. Within the Eurolight project, the sample from Ireland and other samples from Netherlands and Spain were drawn from patient organizations, the samples from Austria, France and United Kingdom were drawn from various clinical settings, and the samples from Germany and Italy were small (*N* < 500), with low participating proportions (< 14%) [36]. In the Fès sub-sample from Morocco, in a breach of protocol, sampling was from random encounters in streets and market places rather than from randomly selected households [24].

All interviews employed the structured HARDSHIP questionnaire [13]. The modular design of HARDSHIP allowed selection of modules to suit each study’s purpose, but all included the demographic (age and gender) and diagnostic modules necessary for our purposes here.

All data included in this meta-analysis were collected between 2008 and 2020.

### Data analysis

We did not register the protocol prior to data analysis since this is not a requirement for IPD meta-analysis of an existing dataset.

#### Headache diagnoses

In the contributing studies, participants were identified as having an active headache disorder if answering “yes” to a neutral screening question (“Have you had a headache in the past year?”) or if they had answered all headache diagnostic questions (which only those with headache in the past year were instructed to do). The diagnostic questions were based on whichever was the current version of the International Classification of Headache Disorders (ICHD) (v2 [45], v3 beta [46] or v3 [47]), and included enquiry into frequency of headache attacks as well as duration, pain characteristics and the presence or not of associated symptoms (nausea, vomiting, photophobia and phonophobia). Changes between these three versions have been inconsequential for the diagnoses of relevance [45–47].

We made only one diagnosis in each participant. In the contributing studies, participants reporting more than one headache type were asked to focus on the most bothersome type when answering the diagnostic questions.

Diagnoses were made by means of the HARDSHIP algorithm [13], which was used in all published Global Campaign studies (Supplementary Fig. 1). This first identified participants with headache on ≥ 15 days/month (H15+), assigning a diagnosis of probable MOH (pMOH) when acute medication was overused and otherwise a label of “other H15+”. The threshold for medication overuse was set to 10 days/month in countries where triptans and/or compound analgesics were considered readily available, on prescription or otherwise (Lithuania, Luxembourg, Morocco, Netherlands, Russia, Saudi Arabia and Spain), and 15 days/month in the others (Benin, Cameroon, Ethiopia, India, Mongolia, Nepal, Pakistan, Peru and Zambia). In the sample from China, we had no information on medication consumption and pMOH could not be diagnosed.

In the standard diagnostic process, the algorithm then applied modified ICHD criteria [13] to all remaining participants (those with headache on < 15 days/month) to diagnose definite migraine, definite TTH, probable migraine or probable TTH, in that order following the ICHD hierarchy [45–47], according to the symptoms and characteristics of the most bothersome headache [13]. Any headache still undiagnosed was unclassified.

In a second (extended) diagnostic process, we applied the algorithm also to those labelled as other H15+ (Supplementary Fig. 2). Hence, migraine or TTH (definite or probable) could be identified among these participants as well as among those with headache on < 15 days/month. Since we had information on frequency of most bothersome headache in addition to frequency of any headache, we were able to apply modified ICHD criteria for chronic migraine (≥ 15 headache days/month, with ≥ 8 migraine days) and chronic TTH (≥ 15 TTH days/month).

These procedures required imputation when crucial diagnostic data were missing. We created neutral or conservative imputation rules that would have the effect of preferring probable rather than definite diagnoses (Table 1). Nevertheless, although nausea and vomiting were imputed as absent when responses were missing on the basis that these bothersome symptoms, when present, were very unlikely to go unreported. Supplementary Table 2 shows the numbers of participants with missing diagnostic information.

**Table 1:** Rules for imputation of missing diagnostic information

Characteristic	When missing, imputed as:	
	applying criteria for migraine	applying criteria for tension-type headache
Duration	4 hours	4 hours
Intensity	moderate/quite bad	moderate/quite bad
Throbbing or pressing	pressing	throbbing
Location	bilateral	unilateral
Aggravation with physical activity	no	yes
Nausea	no	no
Vomiting	no	no
Photophobia	no	yes
Phonophobia	no	yes

#### 1-year prevalence estimation

The 1-year prevalence of any headache was estimated from the total of those answering “yes” to the screening question (numerator) and the total number of participants (denominator). Numerators for each of the different headache types were established by the diagnostic algorithms, with the denominator constant except for pMOH (since pMOH could not be diagnosed in the China sample, the denominator for pMOH excluded this sample). Definite and probable diagnoses were summed to give total prevalence estimates for migraine and TTH.

#### Statistics

Prevalence estimates were reported as proportions (%) with 95% confidence intervals (CIs), separately for each headache type and also stratified by gender, age and country income level. Information from the World Bank relating to the the times of data collection in each country was used to classify countries as low, lower-middle, upper-middle or high income [48]. Since we had limited samples from each level, we grouped high and upper-middle income countries together and low and lower-middle income countries.

We adjusted estimates for age-, gender- and country income level using the most recent (2024) age and gender distributions within each of the four income levels [49] (here we did not dichotomize income level).

We used RStudio version 2023.06.2 + 561 [50] for all analyses. The “geom_smooth”-function (with parameters method=”loess” and formula = y ~ x) within the ggplot package in RStudio was used to plot smoothed prevalence curves against age.

## Results

### Description of the sample

The sample included 41,614 individual participants, 19,336 (46.5%) males and 22,275 (53.5%) females (gender data missing for three). Table 2 shows the basic characteristics of the sample in comparison with those of the world population aged 18–65 years. The mean age was 38.0 (±SD = 13.0) years, similar in males (38.4±13.1) and females (37.6±12.9) (age data missing for 17). Over half of the sample (59.7%) came from lower-middle income countries, 11.4% from upper-middle income, 18.1% from high income, and 10.8% from low income (only the samples from Ethiopia and Nepal).

**Table 2:** Basic characteristics of the sample in comparison with those of the world population aged 18–65 years

	Sample			World [49] (billions)
	Overall	Males	Females	
Sample size, *N* (%)	41,614 (100)	19,336 (46.5)	22,275 (53.5)	4.90 (50.6% males)
Age, mean±SD	38.0±13.0	38.4±13.1	37.7±12.9	-
18–25, *n* (%)	8,764 (21.1)	4,024 (20.8)	4,738 (21.3)	0.98 (20.0)
26–35, *n* (%)	10,719 (25.8)	4,743 (24.5)	5,976 (26.8)	1.19 (24.3)
36–45, *n* (%)	9,964 (23.9)	4,725 (24.4)	5,238 (23.5)	1.08 (22.0)
46–55, *n* (%)	7,031 (16.9)	3,330 (17.2)	3,701 (16.6)	0.92 (18.9)
56–65, *n* (%)	5,119 (12.3)	2,508 (13.0)	2,611 (11.7)	0.73 (14.8)
Income level				
High, *n* (%) Luxembourg, *n* Netherlands, *n* Spain, *n* Saudi Arabia, *n*	7,523 (18.1) 1,825 2,414 968 2,316	3,823 (19.8) 768 1,214 399 1,442	3,700 (16.6) 1,057 1,200 569 874	0.89 (18.2)
Upper-middle, *n* (%) Lithuania, *n* Russia, *n* Peru, *n*	4,746 (11.4) 572 2,025 2,149	2,261 (11.7) 236 960 1,065	2,485 (11.2) 336 1,065 1,084	1.84 (37.6)
Lower-middle, *n* (%) China, *n* Benin, *n* Cameroon, *n* India, Karnataka, *n* India, Delhi NCR, *n* Mongolia, *n* Morocco, *n* Pakistan, *n* Zambia, *n*	24,860 (59.7) 5,041 2,400 3,100 2,329 2,066 2,041 2,575 4,223 1,085	11,334 (58.6) 2,561 1,231 1,407 1,141 737 812 1,038 1,957 450	13,523 (60.7) 2,480 1,169 1,693 1,188 1,329 1,229 1,535 2,265 635	1.85 (37.8)
Low, *n* (%) Ethiopia, *n* Nepal, *n*	4,485 (10.8) 2,385 2,100	1,918 (9.9) 1,057 861	2,567 (11.5) 1,328 1,239	0.31 (6.3)

Compared to the world population aged 18–65 years (in 2024) [49], we had a slight preponderance of females in our sample (53.5% vs. 49.4%), and an overrepresentation of participants from lower-middle income countries (59.7% vs. 37.8%) at the cost of those from upper-middle income countries (11.4% vs. 37.6%). This reflected Global Campaign policy, which focused in its programme of population-based studies on filling the major geographical knowledge gaps [11]. The age distribution was descriptively similar to that of the world population aged 18–65 years, most participants being in their late twenties to early forties and fewest in their late fifties to sixties.

### 1-year prevalence

#### Standard diagnostic process

Table 3 shows the unadjusted estimates for any headache and for each headache type according to the standard diagnostic process, which did not further classify other H15+.

**Table 3:** 1-year prevalence (% [95% CI]) of headache in the general population 18–65 years according to the standard diagnostic process

	Any headache	Migraine			Tension-type headache			H15+	
		Total*	Definite	Probable	Total*	Definite	Probable	pMOH**	Other
Overall	65.5 [65.0–66.0]	24.4 [24.0-24.8]	12.8 [12.5–13.1]	11.7 [11.4–12.0]	32.2 [31.8–32.7]	26.2 [25.8–26.7]	6.0 [5.8–6.2]	4.1 [3.9–4.3]	4.4 [4.2–4.6]
Male	57.9 [57.2–58.6]	18.6 [18.1–19.2]	8.8 [8.4–9.2]	9.9 [9.5–10.3]	33.4 [32.8–34.1]	27.3 [26.7-2]	6.1 [5.8–6.5]	2.3 [2.1–2.5]	3.0 [2.8–3.2]
Female	72.1 [71.5–72.7]	29.5 [28.9–30.1]	16.8 [15.8–16.8]	13.2 [12.7–13.6]	31.2 [30.6–31.8]	25.3 [24.7–25.8]	5.9 [5.6–6.2]	5.6 [5.3–5.9]	5.7 [5.4-6.0]
Age									
18–25	64.5 [63.5–65.5]	22.9 [22.0-23.8]	10.9 [10.3–11.6]	12.0 [11.3–12.7]	34.1 [33.1–35.1]	27.1 [26.2–28.0]	7.0 [6.5–7.6]	2.4 [2.1–2.8]	4.1 [3.7–4.6]
26–35	67.5 [66.6–68.3]	26.0 [25.2–26.8]	13.7 [13.1–14.4]	12.3 [11.6–12.9]	33.2 [32.3–34.1]	26.4 [25.6–27.3]	6.8 [6.3–7.3]	3.6 [3.2–3.9]	4.1 [3.7–4.4]
36–45	67.6 [66.7–68.5]	26.6 [25.8–27.5]	14.4 [13.7–15.1]	12.2 [11.6–12.9]	32.3 [31.4–33.2]	26.7 [25.8–27.5]	5.6 [5.1–6.1]	4.5 [4.0-4.9]	4.2 [3.8–4.6]
46–55	65.5 [64.4–66.6]	24.1 [23.1–25.1]	13.1 [12.3–13.9]	11.1 [10.3–11.8]	30.6 [29.5–31.7]	25.4 [24.3–26.4]	5.3 [4.7–5.8]	5.7 [5.1–6.3]	5.1 [4.6–5.7]
56–65	59.0 [57.6–60.3]	19.8 [18.7–20.9]	10.3 [9.5–11.1]	9.5 [8.7–10.3]	29.0 [27.7–30.2]	24.6 [23.4–25.8]	4.4 [3.8-5.0]	5.6 [4.9–6.4]	5.2 [4.6–5.8]
Income									
High/upper- middle^a^	71.4 [70.6–72.2]	28.5 [27.8–29.3]	16.1 [15.5–16.8]	12.4 [11.8–12.9]	34.6 [33.8–35.5]	27.2 [26.4–28.0]	7.4 [7.0-7.9]	3.9 [3.5–4.2]	3.2 [2.9–3.5]
Low/lowermiddle^b^	63.0 [62.5–63.6]	22.7 [22.3–23.2]	11.4 [11.0-11.7]	11.4 [11.0-11.7]	31.2 [30.7–31.8]	25.8 [25.3–26.3]	5.4 [5.2–5.7]	3.5 [3.3–3.7]	4.9 [4.7–5.2]

*pMOH* probable medication overuse headache, *H15+ *headache on ≥ 15 days/month

^a^Lithuania, Luxembourg, Netherlands, Peru, Russia, Saudi Arabia, Spain [48]

^b^Benin, Cameroon, China, Ethiopia, India (Bangalore and Delhi regions), Mongolia, Morocco, Nepal, Pakistan, Zambia [48]

*definite and probable combined

**China sample excluded (the denominator for pMOH [*N* = 36,573] is therefore different from the denominator for other headache types [*N* = 41,614], and summed prevalences of each type will not perfectly match the prevalence of any headache)

The 1-year prevalence of any headache was 65.5% (95% CI: 65.0–66.0), higher among females (72.1% [71.5–72.7]) than males (57.9% [57.2–58.6]). The most common headache type was TTH (32.2% [31.8–32.7], 26.2% [25.8–26.7] definite, 6.0% [5.8–6.2] probable). Migraine followed (24.4% [24.0-24.8]), but with a more equal split between definite (12.8% [12.5–13.1]) and probable (11.7% [11.4–12.0]). Notably, migraine prevalence among females (29.5% [28.9–30.1]) was almost as high as TTH prevalence (31.2% [30.6–31.8]). The prevalence of pMOH was 4.1% (3.9–4.3) and of other H15 + 4.4% (4.2–4.6). Migraine and H15 + were more prevalent among females than males, whereas TTH was more prevalent among males than females. Only 0.8% (0.7–0.9) of reported headaches remained unclassified, this proportion being similar among males and females.

**Fig. 1:**
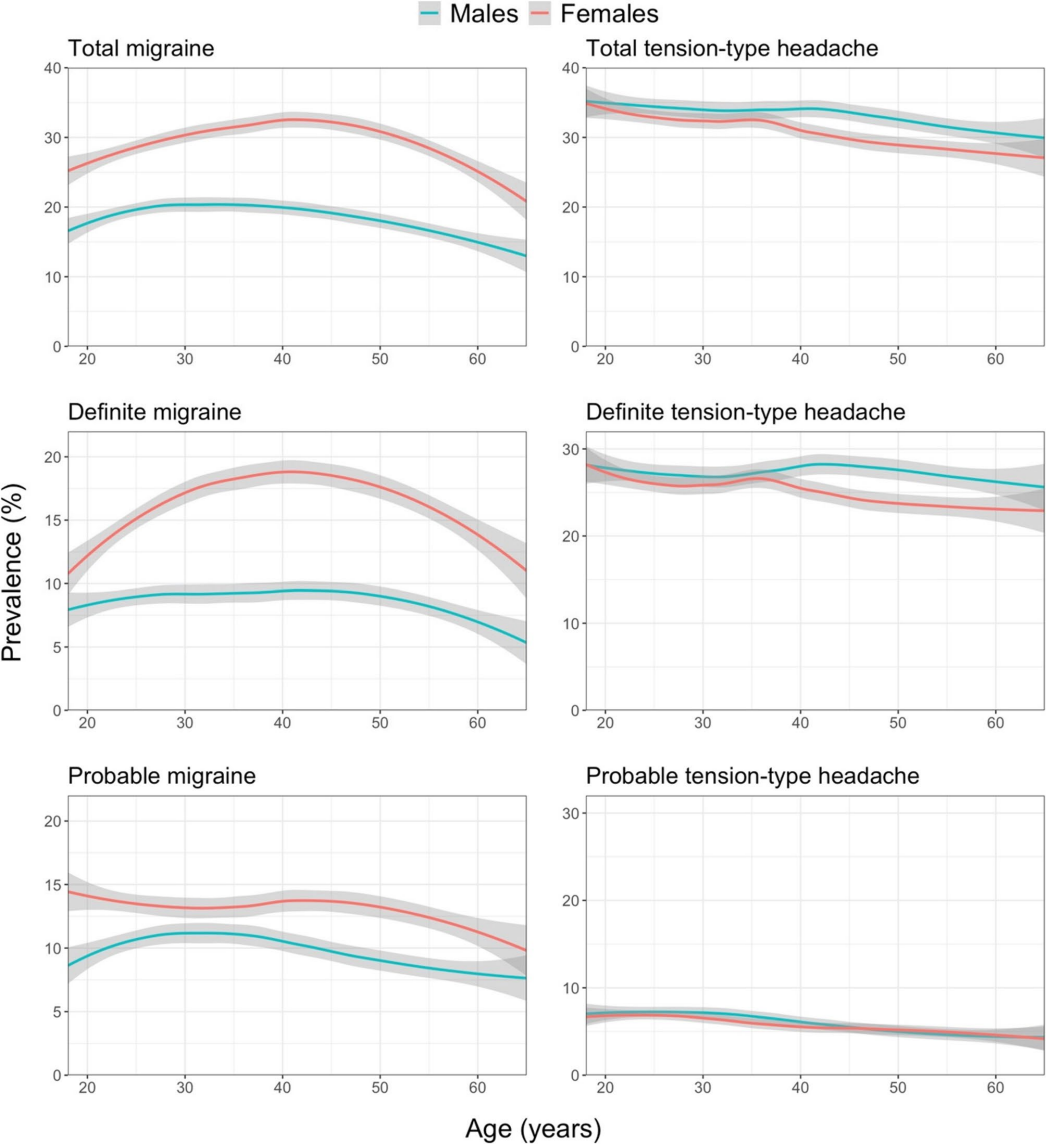
1-year prevalence estimates of migraine and tension-type headache by age and gender according to the standard diagnostic process (grey area: 95% CI of the estimate; the “geom_smooth”-function within the ggplot package in RStudio [with parameters method=”loess” and formula=y~x] was used)

**Fig. 2:**
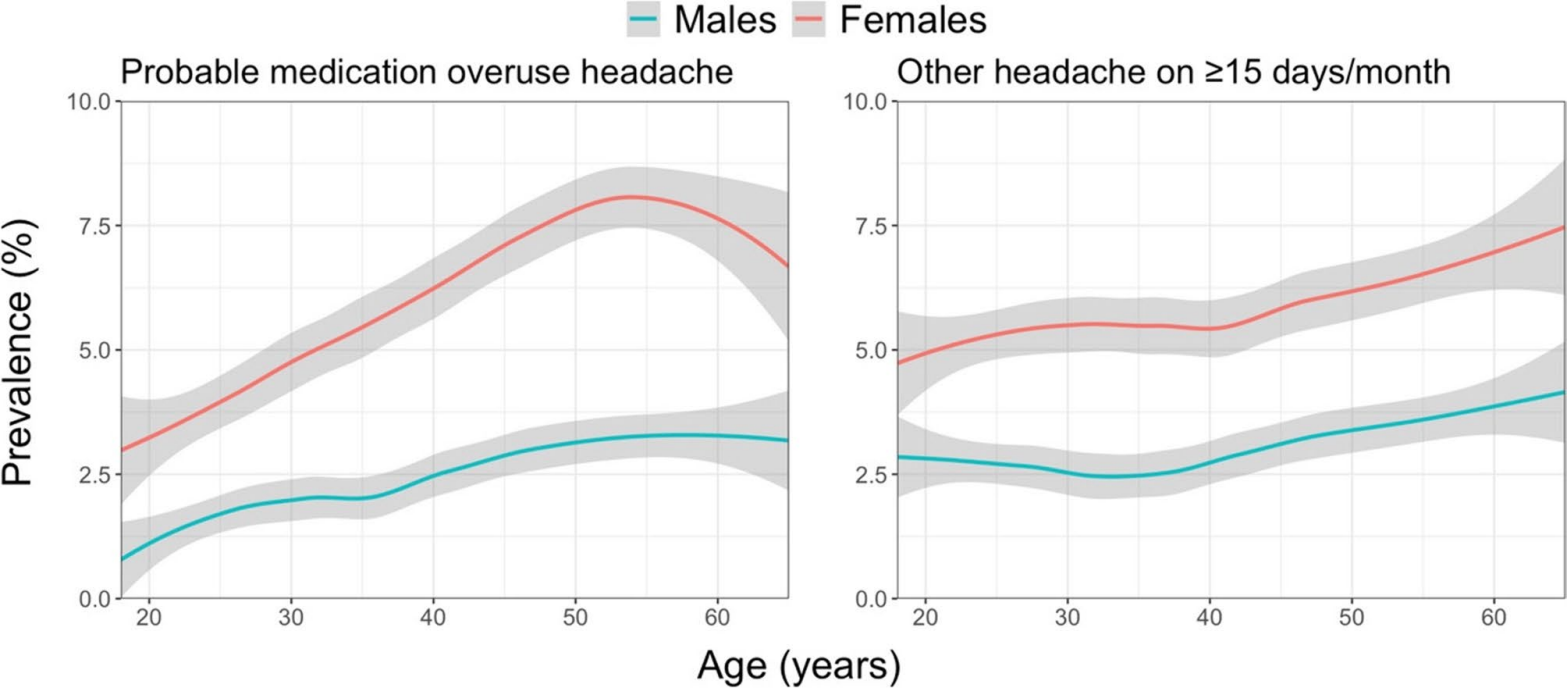
1-year prevalence estimates of headache on ≥15 days/month by age and gender according to the standard diagnostic process (grey area: 95% CI of the estimate; the “geom_smooth”-function within the ggplot package in RStudio [with parameters method=”loess” and formula=y~x] was used)

Table 3 also shows that headache prevalence varied according to age, the relationships being better demonstrated in Figs. 1 and 2. For migraine, there was, clearly, an inverted U-shaped relationship, with prevalence highest in the thirties and forties (30–35% among females, around 20% among males). This inverted U-shape was especially prominent among females with definite migraine; males with migraine showed a flatter curve but still the same relationship. TTH on the other hand, regardless of whether definite or probable, showed a steady slight decline with increasing age among both males and females. pMOH peaked in the fifties at around 8% among females, followed by a sudden decline, whereas, among males, there was a steady increase, with a plateau at 3% in the fifties and sixties. The prevalence of other H15 + increased steadily with age, reaching around 4% among the oldest males and around 7.5% among the oldest females.

Headache prevalence was higher in upper-middle/high income countries (71.4% [70.6–72.2]) than low/lower-middle income countries (63.0% [62.5–63.6]). This was driven by higher prevalences of migraine and TTH.

#### Extended diagnostic process

Table 4 shows how participants with other H15+ (those not reporting medication overuse) were classified according to the symptoms and characteristics of their most bothersome headache. The majority (59.1%) fulfilled the diagnostic criteria for migraine, of whom over half (33.2%) met those for definite migraine; 39.1% fulfilled the criteria for TTH, of whom most (30.1%) met the criteria for definite TTH. Only 1.7% of those with other H15 + remained unclassified.

**Table 4:** Classification of other headache on ≥ 15 days/month by the extended diagnostic process

Diagnosis	Proportion (% [95% CI])
Migraine	59.1 [56.9–61.4]
definite migraine	33.2 [31.1–35.4]
probable migraine	25.9 [23.9–28.0]
Tension-type headache	39.1 [36.9–41.4]
definite tension-tceheeadayp h	30.1 [28.0-32.2]
probable tension-type headache	9.0 [7.8–10.5]
Unclassified headache	1.7 [1.2–2.5]

**Fig. 3:**
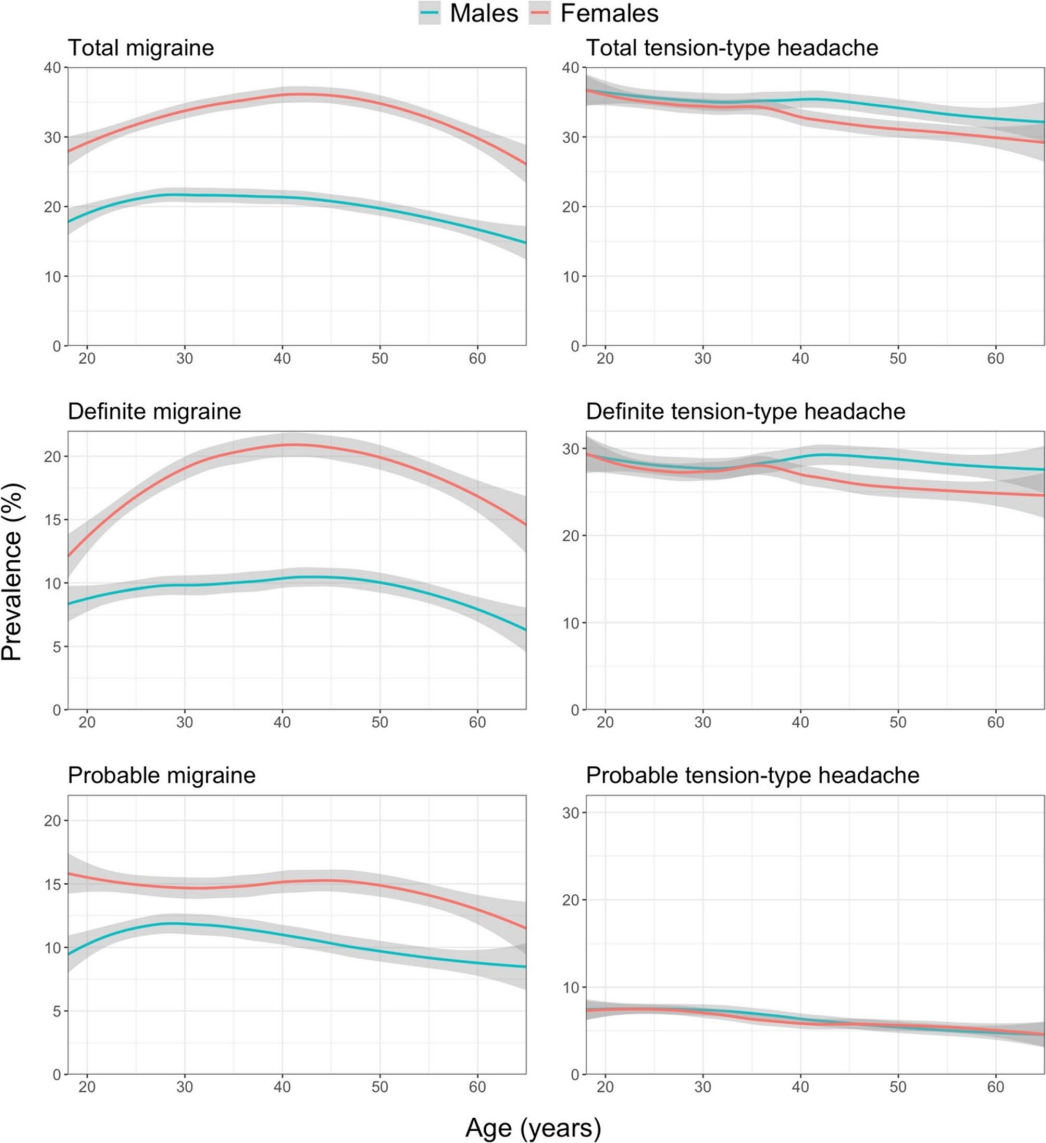
1-year prevalence estimates for migraine and tension-type headache by age and gender according to the extended diagnostic process (grey area: 95% CI of the estimate; the “geom_smooth”-function within the ggplot package in RStudio [with parameters method=”loess” and formula = y ~ x] was used)

Table 5 shows how the prevalence estimates for migraine and TTH increased in the extended diagnostic process applied to other H15+: for migraine from 24.4% (24.0-24.8) to 27.1% (26.6–27.5), and for TTH from 32.2% (31.8–32.7) to 34.0% (33.5–34.4). Relationships with age and gender remained the same (Fig. 3), as did relationships with country income level.

**Table 5:** 1-year prevalence (% [95% CI]) of headache in the general population 18–65 years according to the extended diagnostic process

	**Migraine**			**Tension-type headache**		
	Total*	**Definite**	**Probable**	Total*	**Definite**	**Probable**
Overall	27.1 [26.6–27.5]	14.2 [13.9–14.6]	12.8 [12.5–13.1]	34.0 [33.5–34.4]	27.6 [27.1–28.0]	6.4 [6.2–6.6]
Male	20.1 [19.6–20.7]	9.5 [9.1–10.0]	10.6 [10.2–11.0]	34.9 [34.2–35.5]	28.4 [27.8–29.0]	6.4 [6.1–6.8]
Female	33.1 [32.5–33.7]	18.3 [17.8–18.8]	14.7 [14.3–15.2]	33.2 [32.6–33.8]	26.8 [26.2–27.4]	6.4 [6.0-6.7]
Age						
18–25	25.3 [24.4–26.2]	12.0 [11.4–12.7]	13.2 [12.5–13.9]	35.8 [34.8–36.8]	28.3 [27.4–29.2]	7.5 [6.9-8.0]
26–35	28.4 [27.5–29.2]	15.0 [14.3–15.7]	13.4 [12.7–14]	34.9 [34.0-35.8]	27.6 [26.8–28.5]	7.2 [6.7–7.7]
36–45	29.3 [28.4–30.2]	16.0 [15.3–16.7]	13.3 [12.6–13.9]	33.8 [32.9–34.8]	28.0 [27.1–28.9]	5.9 [5.4–6.3]
46–55	27.1 [26.1–28.1]	14.8 [14.0-15.6]	12.3 [11.5–13.1]	32.7 [31.6–33.8]	26.9 [25.8–27.9]	5.8 [5.3–6.4]
56–65	23.0 [21.8–24.1]	12.2 [11.3–13.1]	10.7 [9.9–11.6]	30.9 [29.7–32.2]	26.2 [25-27.4]	4.7 [4.1–5.3]
Income						
High/upper- middle^a^	30.4 [29.6–31.2]	17.2 [16.5–17.9]	13.2 [12.6–13.8]	35.8 [35.0-36.7]	28.0 [27.3–28.8]	7.8 [7.3–8.3]
Low/lowermiddle^b^	25.7 [25.2–26.2]	13.0 [12.6–13.4]	12.6 [12.3–13.0]	33.2 [32.7–33.7]	27.4 [26.8–27.9]	5.8 [5.6–6.1]

*pMOH* probable medication overuse headache, *H15+ *headache on ≥ 15 days/month

^a^Lithuania, Luxembourg, Netherlands, Peru, Russia, Saudi Arabia, Spain [48]

^b^Benin, Cameroon, China, Ethiopia, India (Bangalore and Delhi regions), Mongolia, Morocco, Nepal, Pakistan, Zambia [48];

*definite and probable combined

#### Chronic migraine and chronic tension-type headache

The tentatively estimated 1-year prevalence of chronic migraine was 1.7% (1.5–1.8), higher in females (2.2% [2.0-2.4]) than males (1.0% [0.9–1.1]), and of chronic TTH was 0.9% (0.8-1.0), also higher in females (1.0% [0.9–1.2]) than males (0.7% [0.6–0.9]).

### Age-, gender- and income-adjusted global estimates

Table 6 shows the estimated global 1-year prevalence of each of the principal headache types when adjusted for age, gender and country income level. According to the standard diagnostic process, an estimated 65.0% (64.5–65.5) of the global population aged 18–65 years had an active headache disorder (at least one headache episode during the preceding year). The largest proportion had TTH (33.2% [32.7–33.6]), but very many had migraine (23.5% [23.1–23.9]), about half of whom fulfilled the criteria for definite migraine (12.8% [12.4–13.1]). In total, 8.1% had H15+, 4.1% (3.9–4.3) with associated medication overuse (pMOH). The extended diagnostic process increased the migraine estimate to 25.9% (25.4–26.3), and the TTH estimate to 34.7% (34.3–35.2).

**Table 6:** 1-year global headache prevalence estimates according to each diagnostic process, adjusted for age, gender and country income level (% [95% CI])

Headache type	Standard diagnostic process	Extended diagnostic process
Any headache	65.0 [64.6–65.5]	65.0 [64.6–65.5]
Migraine	23.5 [23.1–23.9]	25.9 [25.4–26.3]
definite migraine	12.8 [12.4–13.1]	14.1 [13.8–14.5]
probable migraine	10.8 [10.5–11.1]	11.7 [11.4–12.0]
Tension-type headache	33.2 [32.7–33.6]	34.7 [34.3–35.2]
definite tension-type headache	27.5 [27.1–28.0]	28.8 [28.3–29.2]
probable tension-type headache	5.6 [5.4–5.9]	6.0 [5.8–6.2]
Probable medication overuse headache*	4.1 [3.9–4.3]	4.1 [3.9–4.3]
Other headache on ≥ 15 days/month	4.0 [3.8–4.2]	-
Unclassified headache	0.7 [0.6–0.8]	0.8 [0.7–0.8]

* China sample excluded (the denominator for pMOH [*N* = 36,573] is therefore different from the denominator for other headache types [*N* = 41,614], and summed prevalences of each type will not perfectly match the prevalence of any headache)

The adjusted tentative estimates for chronic migraine and chronic TTH were 1.5% (1.3–1.6) and 0.8% (0.7–0.9) respectively.

### Supplementary analyses

Of the 10,166 diagnosed with migraine by the standard diagnostic process, 3,239 reported more than one headache type (1,003 males, 2,236 females); of the 13,414 diagnosed with TTH, 3,134 reported more than one type (1,386 males, 1,747 females; gender data missing for one). Of the 11,259 diagnosed with migraine by the extended diagnostic process, 3,615 reported more than one headache type (1,099 males, 2,516 females); of the 14,137 diagnosed with TTH, 3,355 reported more than one type (1,469 males, 1,885 females; gender data missing for one).

Since headache prevalence varied with age and gender, and the distribution of both these variables differed between the low/lower-middle income countries (mean age = 37.4 years, proportion of males = 45.2%) and high/upper-middle income countries (mean age = 39.6 years, proportion of males = 49.6%), we investigated the relationship between headache prevalence and country income level. Adjusted for age and gender, the 1-year prevalence of any headache was 62.4% (62.0-62.9) in low/lower-middle income countries and 71.1% (70.7–71.6) in high/upper-middle income countries, both estimates similar to the observed prevalences. As a sensitivity analysis, we excluded China (the sole outlier of the contributing studies), which raised the observed prevalence of any headache in the low/lower-middle income countries to 70.5% (69.9–71.1), very similar to the 71.4% observed in high/upper-middle income countries.

**Fig. 4:**
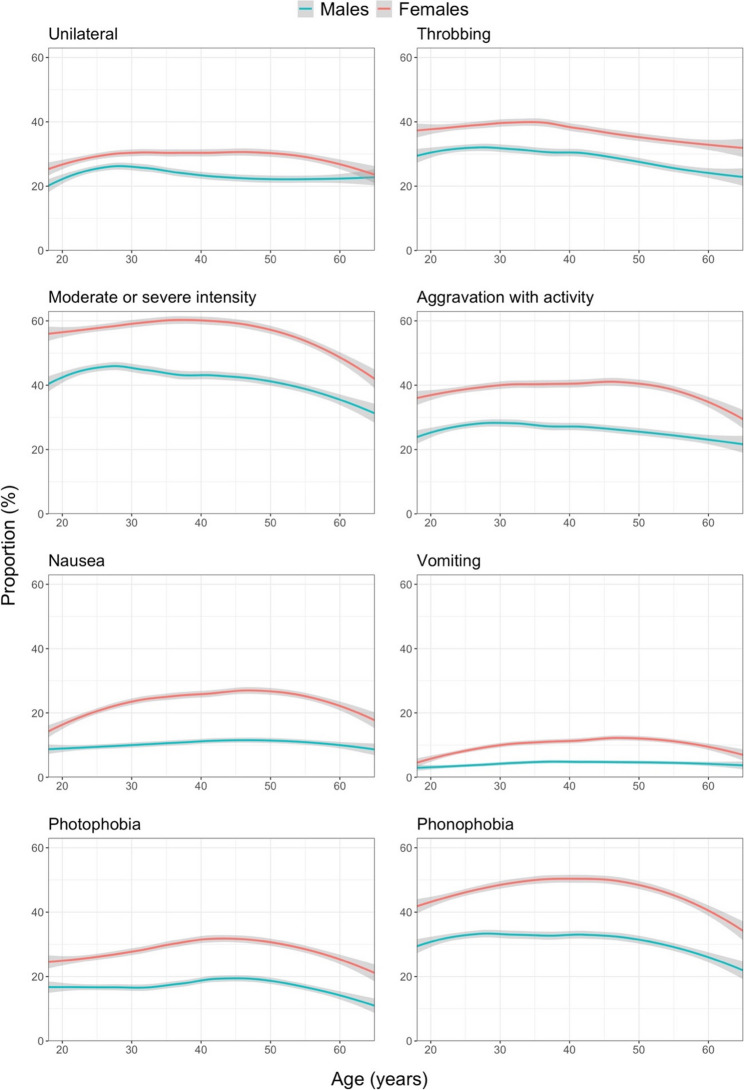
Proportions of all participants reporting migraine-specific characteristics by age and gender (grey area: 95% CI of the estimate; the “geom_smooth”-function within the ggplot package in RStudio [with parameters method=”loess” and formula = y ~ x] was used)

In an endeavour to understand the inverted U-shaped relationship between age and migraine prevalence, the proportions of all participants reporting headache characteristics specific for migraine were plotted against age (Fig. 4). All showed a similar inverted U-shaped pattern, especially among females.

As mentioned, Supplementary Table 2 shows the numbers of participants with missing diagnostic information, where imputation was used. Of those with any headache, 8.9% were missing data in one or more of the diagnostic question fields. The highest proportion was among those whose headache remained unclassified (54.8%); the lowest were for definite migraine (7.2%) and definite TTH (5.8%). Duration was most often missing (4.3%), followed by photophobia (2.9%). Very few participants (0.5%) were missing data in all diagnostic fields; most of those who were remained unclassified, with none diagnosed as migraine or TTH (definite or probable), a reflection of our imputation rules.

## Discussion

This large meta-analysis pooling individual participant data from population-based studies in 17 countries from all parts of the world found that most adults of working age report an active headache disorder. Using two diagnostic processes, and adjusting for age, gender and country income level, we estimate that 65.0% of the world’s population aged 18–65 years will have headache in the coming year; in about half (33.2–34.7%) this will be TTH, but very many will have migraine (23.5–25.9%). Of considerable concern, 4.1% will have pMOH.

Before discussing or attempting to interpret these results, we need to scrutinize the methodological limitations. The standard diagnostic process allowed migraine and TTH to be diagnosed only among participants with headache on < 15 days/month, effectively excluding chronic migraine and chronic TTH from prevalence estimates for migraine and TTH. All Global Campaign studies adopted this approach, the reasons being two-fold. First, diagnosis of H15 + is challenging even in clinic, and rarely possible at a single encounter. In clinic, the diagnosis needs to be correct, and is established during follow-up, whereas epidemiological studies, unable to do this, can accept a level of probability. Second, secondary headaches are more likely to present as H15 + than as recurrent episodic headache. For obvious reasons, epidemiological questionnaire-based enquiry cannot reliably evaluate ICHD diagnostic criterion E (not better accounted for by another ICHD diagnosis), which might identify secondary headaches. The extended diagnostic process allowed migraine and TTH to be diagnosed regardless of headache frequency, essentially trading specificity for sensitivity with the possibility of incorrectly identifying secondary headaches as migraine or TTH. But, except for MOH, chronic secondary headaches are rare (< 1%) in the adult general population [51], especially among those fit and willing to respond to an impromptu survey.

We therefore believe the extended diagnostic process provides estimates of migraine and TTH that are closer to the truth than those produced by the standard process, since the latter clearly, and erroneously, excludes the chronic subtypes of migraine (1.6%) and TTH (0.9%), as well as the 1.5% with H15 + who probably had concomitant episodic migraine and episodic TTH. The extended diagnostic process is probably also more in accordance with how ICHD should be applied. We present estimates from both processes, emphasizing that they are *estimates*. While the differences were no more than 2–3% points for migraine and TTH, the impact on *burden* estimates (largely based on time with headache) will be much greater. These estimates are pending in future publications.

Another limitation of importance in our diagnostic process was that we were only able to diagnose one headache type per participant, the most bothersome type when more than one type was reported. This certainly led to underestimation of TTH, since those with TTH and concomitant migraine were far more likely to regard the latter as more bothersome. Our supplementary analysis showed that 3,615 of those diagnosed with migraine (8.7% of the entire sample) according to the extended diagnostic process reported more than one headache type. Of these, 2,516 (69.6%) were females. We can only speculate with regard to what the other type(s) might be. While more than one subtype of migraine is possible (migraine with and without aura), it is questionable whether participants in these surveys would distinguish these. Much more likely was TTH, given the high prevalence of TTH (almost 30%). While a similar proportion of those with TTH also reported more than one headache type (3,355; 8.1% of the entire sample), probably very few of these had migraine, without perceiving this as their most bothersome headache. The characteristics of TTH are highly variable, and more than one TTH-like headache is possible, but, since these participants were already diagnosed with TTH, this would not add to TTH prevalence estimates.

**Fig. 5:**
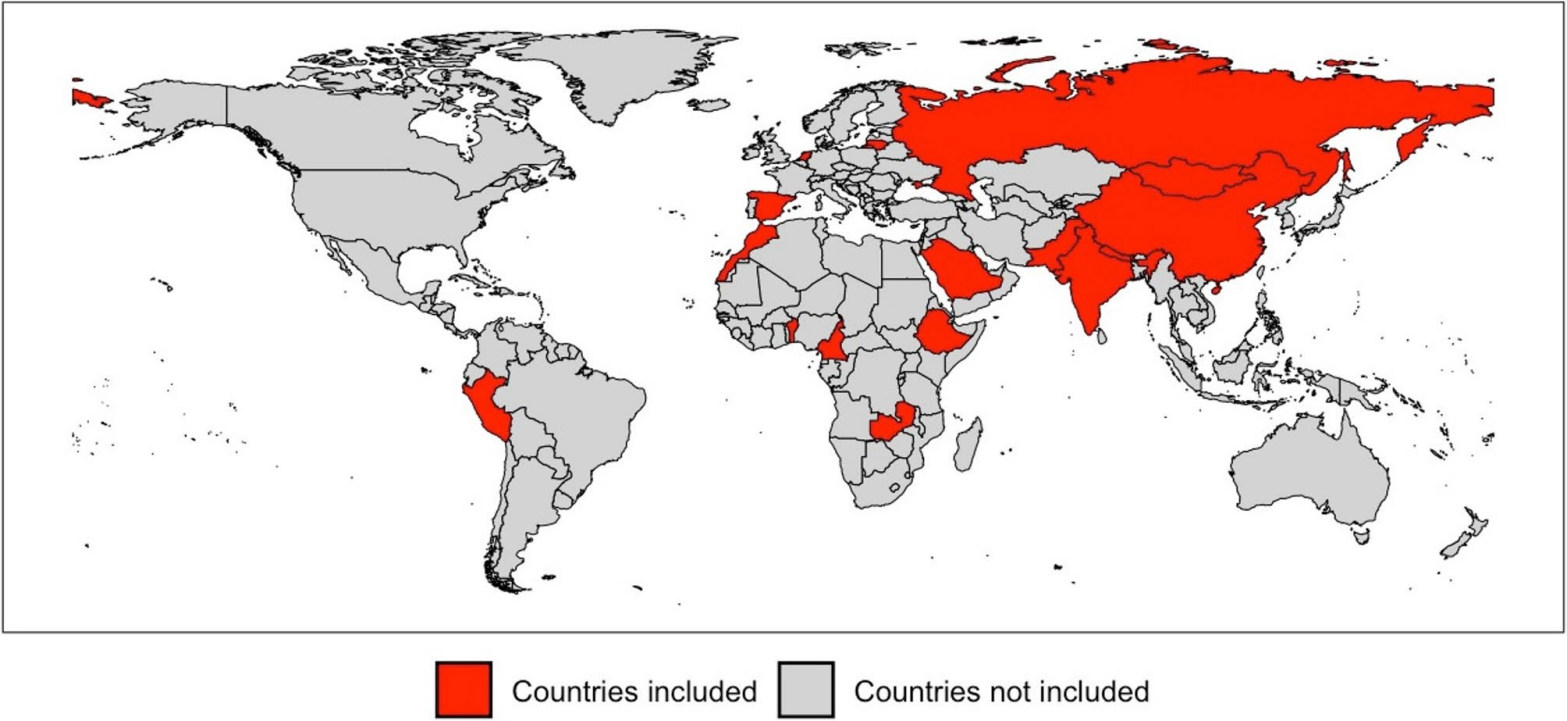
Map of the world showing the 17 countries (in red) included in our meta-analysis

A second methodological limitation is in how the present meta-sample was derived, and how representative it is of the world’s population aged 18–65 years. It is constituted of sub-samples from 17 countries from all parts of the world, themselves derived through cluster-based random sampling in order to be representative of the general populations being sampled. This, we argue, represented a cluster sampling of the world, with correction for age, gender and country income level methodologically appropriate. In not all cases were the sub-samples of national populations, but this meant only that the cluster was smaller than the country. Neither, and perhaps more importantly, were the countries themselves randomly selected, but chosen largely with regard to feasibility, given that the underlying aim of the Global Campaign had been to undertake studies in parts of the world where none or few had been performed previously [52]. This is very evident in Fig. 5, and led to overrepresentation of lower-middle income countries, necessitating adjustment for country income level as well as for age and gender.

The third methodological limitation underlies all epidemiological headache surveys: how the ICHD criteria behave in different populations. ICHD was developed by experts applying knowledge that came, in the main, from high-income western countries. Since the criteria for primary headaches depend wholly on subjective reporting of headache characteristics, language and culture inevitably have influence. In Nepal, for example, photophobia was reported by virtually every participant with headache, rendering it useless in the differentiation between migraine and TTH [25]. Disregarding this (treating it as missing in all cases) was the only country-specific variation in the diagnostic algorithm in this present study.

A fourth methodological consideration (arguably not a limitation) was our use of imputation to replace missing data, an element in all large datasets. Exclusion of participants reporting headache but with missing diagnostic data would lead to underestimation of overall headache prevalence: here, it would have reduced the participants with headache from 27,257 (65.5%) to 24,827 (59.7%). Neutral and/or conservative imputation, leading towards probable rather than definite diagnoses, and towards unclassified headache, was, we believe, the most reasonable approach.

### Prevalence related to age and gender

Unsurprisingly, headache was more common among females than males. This was true for migraine (1.5-fold difference) and pMOH (more than 2-fold difference). However, over the years the reported gender-difference in estimates of migraine prevalence has decreased from 3-fold [52–54] to 2-fold [55, 56]. Our finding of even less difference, from studies of high quality and with population-representative samples, continues this trend.

TTH was slightly more common among males (34.9%) than females (33.2%). This contrasts with GBD, which finds TTH to be slightly more common among females (25.6% vs. 23.9%) [5, 56]. Since we were not able to identify TTH in those with concomitant migraine (see above), since migraine was more prevalent among females, and since, of those diagnosed with migraine and reporting a second headache type, 69.6% were female (also see above), we almost certainly underestimated TTH by a greater margin among females than among males. GBD includes some studies reporting only the prevalence of TTH, which would not suffer from this limitation.

Albeit more pronounced in females, both genders displayed the same age-relationship for migraine: a clear inverted U-shape, with a peak in the thirties to forties, which was mostly driven by definite migraine. The supplementary analyses showed that this was not explained by any one of the migraine characteristics – rather, all characteristics contributed more or less equally. This suggests that migraine has similar characteristics at all ages. There is an element of self-fulfilment in this, since migraine is defined by these characteristics. It is nevertheless a question that future studies should investigate.

The prevalence of pMOH increased with age, peaking in the mid-fifties, which is logically explained by the time (often years) it takes for MOH to develop, through overconsumption of medication, from a primary headache that first must be acquired (both migraine and TTH have age-related onset). The sharp decrease in prevalence seen among females after their mid-fifties is probably a reflection of remission of the underlying primary headache, in most cases migraine [7].

In contrast to migraine and pMOH, TTH was most prevalent among the youngest participants, becoming slightly less common with increasing age.

### Prevalence related to country income level

We found headache overall to be more common in high/upper-middle income countries than in low/lower-middle income countries. This aligns with GBD [56]. Importantly, we had limited representation from the low income level (only Ethiopia and Nepal), and chose therefore to classify by two levels only, losing granularity. Our relatively low estimate of prevalence in low/lower-middle income countries was attributable to the outlying and low estimates for all headache types in China [18], and could not be explained by differences in age and gender distribution between the two income levels (see supplementary analyses). The study in China was of high quality, with a sample well representative of the general population and interviews conducted by neurologists [33], so we have no reason to question these estimates, but their influence was large because our analysis used IPD and the sample size in China was large (*N* = 5,041). Other contributing studies mostly had *N* ≈ 2,000, although India, the other highly populous country (lower middle income), had *N* = 4,395 from two studies [57, 58]). Since China’s population is about 17.5% of the global population, its influence on our estimates was not untoward (we made no other adjustment for country population size), but we do not know whether China was an outlier because of biology or a culturally determined disinclination to report headache. Supporting the latter view is previous research speculating that headache is perceived as an emotional issue or a sign of weakness in China, and is therefore often unacknowledged, particularly among men [18, 59]. A literature review from 2019 highlighted low disease awareness of headache in East Asian countries, and that many people with symptoms do not consult physicians [60].

The prevalence of pMOH was similar for the two income levels, but important to this finding is that we did not have information on medication consumption in China, which was therefore excluded from the pMOH analysis (reducing the denominator to *N* = 36,573). Also important is that we applied different thresholds for medication overuse (10 or 15 days/month) in the various samples according to the drugs that were widely available, a factor likely to have aligned with country income level. This may have introduced a degree of bias: overestimation of pMOH – among participants using only simple analgesics despite availability of triptans – was more probable in higher-income countries. pMOH comparisons between the two income levels therefore require caution. Recognizing these limitations, we can say only that living in a low/lower-middle income country appears to be no hindrance to acute medication overuse, which entails the risk of developing MOH.

Since pMOH could not be diagnosed in China, all participants in this country reporting H15 + were classified as other H15+. However, this cannot explain the higher prevalence of other H15 + in low/lower-middle income countries: the prevalence of H15 + in China (1.0% [18]) was considerably lower than the estimate for high/upper-middle income countries. Possibly, it is indicative of more secondary headache in low/lower-middle income countries.

### Comparison with previous global estimates

We found higher prevalences than other recent studies. This was true for migraine (25.9% vs. 14–15% [1–5]), TTH (34.7% vs. 26% [2–5]) and MOH (4.1% vs. 1–2% [6–9]). Partly this can be explained by the narrower age range in our sample (18–65 years), excluding children and the elderly. The online GBD compare tool [56], with adults aged 20–54 years selected (the most comparable age group), yields a global migraine prevalence of 20.6% [56], still lower than but much closer to our estimate (25.9%), and a TTH prevalence of 32.5% [56], quite similar to ours (34.7%).

However, the age associations in our findings show that our estimates would have been even higher for those aged 20–54 rather than 18–65 years. Further, GBD methodology [5] attributes pMOH to migraine or TTH in the ratio 73.2:26.8, as presumed sequelae of these disorders. If we had done the same, our prevalence estimates for migraine and TTH would have been higher by at least 1–2% points. And, as previously discussed, our TTH estimate was too low since only the most bothersome headache type was diagnosed. This limitation did not apply to all studies informing GBD.

Therefore, our estimates of global migraine prevalence are considerably higher than those from GBD, and this still requires explanation. GBD includes many more samples, with much variation in methodology. We have previously shown that case definition greatly influences prevalence estimates [2], particularly the inclusion of probable diagnoses, which our contributory studies did but many of those included in GBD did not (although GBD endeavours to correct for this using regression models). Other methodological variations are easily accounted for, in particular the screening question. All our contributing studies used the same neutral screening question (“have you had headache in the last year”); other studies have imposed limiting descriptors, such as “severe headache”, or “headache not due to other causes”. Publication year is also a factor: higher estimates are associated with more recent publications [2], probably because of refinements in methodology over the years. All of our contributory studies, published from 2012 onwards, were performed with the same instrument and the same methodology [10, 13], negating this influence, but GBD includes many older studies.

In conclusion, we believe our estimates of the current global prevalences of migraine and pMOH in the general population aged 18–65 are more reliable and accurate than GBD estimates. For reasons discussed, our estimate for TTH was probably substantially too low.

### Strengths and limitations

The principal strengths of this meta-analysis were the use of IPD and the very large number of participants (*N* = 41,614), all from relatively recent population-based studies based on the same standardized methodology [10, 13]. Pooled IPD are considered the gold standard for meta-analyses [14]. The large sample enabled granular scrutiny and analytical flexibility (evidenced by supplementary analyses), with high statistical power. Also a strength was that we excluded samples not meeting rigorous criteria for general-population representativeness (including several of the Eurolight samples [36] and the Fès subsample from Morocco [24]). We used neutral or conservative imputation when diagnostic information was missing, rather than exclusion.

Limitations were also present, some of which have already been discussed at length. The design of all contributing studies was cross-sectional, with reliance on participants’ reporting of past symptoms and vulnerability to recall error. The cross-sectional designs also required modifications of ICHD criteria [13]: criterion A (a history of multiple attacks) was assumed to be met when participants reported headache in the past year, although frequency reporting almost invariably provided confirmation of this. Criterion E (not better accounted for by another ICHD diagnosis), however, could not be reliably ascertained. Nonetheless, the standard algorithmic diagnostic process has been validated in six studies [33, 40, 57, 58, 61, 62]. The extended process has not, and the argument here that it is preferable requires further empirical assessment. Our diagnoses of chronic migraine and chronic TTH using the extended process are tentative, not least because they are not informed by 3-month histories. The lack of information on medication consumption in the China sample reduced the denominator for pMOH estimates to *N* = 36,573.

## Conclusions

Two thirds (an estimated 65%) of the global population aged 18–65, who are likely to include most of the world’s workforce, will suffer from headache during the coming year. Around 25% will have migraine and, of considerable concern, over 4% will have pMOH. These last two estimates are higher than previous comparable estimates, and, notably, with smaller gender-differences (1.5-fold for migraine). We believe these estimates to be the best currently available for this population. Our estimate of TTH prevalence (over 33%) is probably substantially too low, since methodological constraints precluded identification of TTH in those with concomitant migraine.

Future analyses will be of the global burden associated with these headache disorders.

## Supplementary Information

Supplementary Material 1: Supplementary Figure 1. Flowchart of the standard diagnostic process. Supplementary Figure 2. Flowchart of the extended diagnostic process. Supplementary Table 1. Design and characteristics of studies included in the adult Headache-Attributed Restriction, Disability, Social Handicap and Impaired Participation (HARDSHIP) database. Supplementary Table 2. Numbers of participants with missing diagnostic information.

## Data Availability

The original data are held at Norwegian University of Science and Technology, Trondheim, Norway. Anonymised data are available on request for academic purposes, in line with the policy of the Global Campaign against Headache.

## Abbreviations

CI: Confidence interval
GDB: Global Burden of Disease
HARDSHIP: Headache-attributed restriction, disability, social handicap and impaired participation (questionnaire)
H15+: Headache on ≥15 days/month
ICHD: International Classification of Headache Disorders
LTB: *Lifting The Burden*
MOH: Medication-overuse headache
NCR: North capital region (of India)
NorHEAD: Norwegian Centre for Headache Research
NTNU: Norwegian University of Science and Technology
pMOH: Probable MOH
SD: Standard deviation
TTH: Tension-type headache
