# Supplementary material for: The global prevalence of headache disorders of public-health importance: a meta-analysis of population-based individual participant data from 41,614 adults from 17 countries
Source: J Headache Pain. 2025 Oct 7;26(1):204. doi: 10.1186/s10194-025-02142-9 (PMC12502191; doi:10.1186/s10194-025-02142-9)
Supplement: Supplementary file 1 — Supplementary Material 1: Supplementary Figure 1. Flowchart of the standard diagnostic process. Supplementary Figure 2. Flowchart of the extended diagnostic process. Supplementary Table 1. Design and characteristics of studies included in the adult Headache-Attributed Restriction, Disability, Social Handicap and Impaired Participation (HARDSHIP) database. Supplementary Table 2. Numbers of participants with missing diagnostic information. [file 10194_2025_2142_MOESM1_ESM.docx]

#### Supplementary Figure 1. Flowchart of the standard diagnostic process

*Medication overuse defined as acute medication on ≥10 days/month in Lithuania, Luxembourg, Morocco, Netherlands, Russia, Saudi Arabia and Spain and as ≥15 days/month in Benin, Cameroon, Ethiopia, India, Mongolia, Nepal, Pakistan, Peru and Zambia.

**Diagnosed in accordance with ICHD in the hierarchical order shown from top to bottom (*ie* definite migraine first, unclassified last).

#### Supplementary Figure 2. Flowchart of the extended diagnostic process

*Medication overuse defined as acute medication on ≥10 days/month in Lithuania, Luxembourg, Morocco, Netherlands, Russia, Saudi Arabia and Spain and as ≥15 days/month in Benin, Cameroon, Ethiopia, India, Mongolia, Nepal, Pakistan, Peru and Zambia.

**Diagnosed in accordance with ICHD in the hierarchical order shown from top to bottom (*ie* definite migraine first, unclassified last).

#### Supplementary Table 1. Design and characteristics of studies included in the adult Headache-Attributed Restriction, Disability, Social Handicap and Impaired Participation (HARDSHIP) database

| **Study** | **Data collection (year)** | **Underlying population** | **Sampling method** | **Sample size** | **Participating proportion** | **Engagement** | **Interviewers** | **Included in this meta-analysis** |
| --- | --- | --- | --- | --- | --- | --- | --- | --- |
| Austria [36] | 2009 | Clinic-based, patients consulting GPs or neurologists for any reason | Random sample uncorrected for demographics | 519 | N/A | Self-administered questionnaire | None, handed questionnaire at consultation | No |
| Benin [16] | 2020 | General population from whole country | Random sample corrected for demographics | 2,400 | 94.1% | Face-to-face interviews | Trained interviewers with medical/research background | Yes |
| Cameroon [17] | 2019 | General population from whole country | Random sample corrected for demographics | 3,100 | 93.3% | Face-to-face interviews | Trained interviewers with medical/research background | Yes |
| China [33] | 2008-2009 | General population from whole country | Random sample corrected for demographics | 5,041 | 94.1% | Face-to-face interviews | Neurologists | Yes |
| Ethiopia [19] | 2014 | General population from whole country | Random sample corrected for demographics | 2,385 | 99.8% | Face-to-face interviews | Trained nurses | Yes |
| France [36] | 2009 | Clinic-based, patients consulting GPs for any reason | Random sample uncorrected for demographics | 680 | 36.5% | Self-administered questionnaire | None, handed questionnaire at consultation | No |
| Germany [36] | 2009 | General population from part of the country | Random sample corrected for demographics | 318 | 10.6% | Self-administered questionnaire | None, contacted by regular post | No |
| India, Delhi region [57] | 2018-2019 | General population from part of the country | Random sample corrected for demographics | 2,066 | 67.9% | Face-to-face interviews | Trained research officers | Yes |
| India, Karnataka state [58] | 2009 | General population from part of the country | Random sample corrected for demographics | 2,329 | 92.6% | Face-to-face interviews | Trained research officers | Yes |
| Ireland [36] | 2009 | Members of patient organization | Total defined population | 225 | N/A | Self-administered questionnaire | None, handed questionnaire at consultation | No |
| Italy [36] | 2009 | General population from part of the country | Stratified (age, sex, habitation), random sample | 487 | 13.9% | Self-administered questionnaire | None, contacted by regular post | No |
| Lithuania [21] | 2009-2010 | General population from part of the country | Stratified (age, habitation), random sample | 572 | 50.4% | Face-to-face interviews | Trained medical students | Yes |
| Luxembourg [36] | 2008-2009 | General population from whole country | Stratified (age, gender, habitation), random sample | 1,825 | 28.2% | Self-administered questionnaire | None, contacted by regular post | Yes |
| Mali [44]* | 2021 | General population from whole country | Random sample corrected for demographics | 2,105 | 99.4% | Face-to-face interviews | Trained interviewers | No |
| Mongolia [23] | 2017 | General population from whole country | Random sample corrected for demographics | 2,041 | 98.3% | Face-to-face interviews | Trained neurologists | Yes |
| Morocco (population sample) [24] | 2019 | General population from whole country | Random sample corrected for demographics | 2,575 | 90.0% | Face-to-face interviews | Trained medical students | Yes |
| Morocco (Fes sample) [24] | 2019 | General population from part of the country | Random sample not corrected for demographics | 899 | N/A | Face-to-face interviews | Trained medical students | No |
| Nepal [25] | 2013 | General population from whole country | Random sample corrected for demographics | 2,100 | 99.6% | Face-to-face interviews | Trained health workers | Yes |
| Netherlands (patient sample) [36] | 2009 | Members of patient organization | Random sample not corrected for demographics | 430 | N/A | Self-administered questionnaire | None, handed questionnaire at consultation | No |
| Netherlands (population sample) [36] | 2010 | General population from whole country | Stratified (age, gender, habitation, education social status), random sample | 2,414 | N/A | Self-administered questionnaire | None, contacted through internet | Yes |
| Pakistan [40] | 2010 | General population from whole country | Random sample corrected for demographics | 4,223 | 98.5% | Face-to-face interviews | Trained non-medical interviewers | Yes |
| Peru [27] | 2019 | General population from whole country | Random sample corrected for demographics | 2,149 | 90.1% | Face-to-face interviews | Trained health workers | Yes |
| Russia [28] | 2008 | General population from whole country | Random defined sample | 2,025 | 74.3% | Face-to-face interviews | Trained non-medical interviewers | Yes |
| Saudi Arabia [29] | 2012 | General population from whole country | Random defined sample | 2,316 | 86.5% | Telephone interviews | Trained research coordinators with health background | Yes |
| Spain (patient sample) [36] | 2009 | Members of patient organization | Random sample uncorrected for demographics | 264 | 58.8% | Self-administered questionnaire | None, handed questionnaire at consultation | No |
| Spain (workplace sample) [36] | 2009 | Workplace: postal service employees | Total defined population | 968 | 58.8% | Self-administered questionnaire | None, contacted by internal post | Yes |
| United Kingdom [36] | 2009 | Clinic-based, patients consulting GPs for any reason | Random sample uncorrected for demographics | 99 | 17.6% | Self-administered questionnaire | None, handed questionnaire at consultation | No |
| Zambia [43] | 2012 | General population from whole country | Random sample corrected for demographics | 1,085 | 95.7% | Face-to-face interviews | Trained health workers | Yes |

*Lacking diagnostic information necessary to identify migraine or tension-type headache.

#### Supplementary Table 2. Numbers of participants with missing diagnostic information

|  | **Any headache** | **Migraine** | | | **TTH** | | | **pMOH** | **Other H15+** | **Unclassified** |
| --- | --- | --- | --- | --- | --- | --- | --- | --- | --- | --- |
|  |  | Total | Definite | Probable | Total | Definite | Probable |  |  |  |
| Duration | 1161  (4.3%) | 346  (3.4%) | 233  (4.4%) | 113  (2.3%) | 469  (3.5%) | 442  (4.1%) | 27  (1.1%) | 60  (4.0%) | 166  (9.0%) | 120  (36.1%) |
| Intensity | 276  (1.0%) | 38  (0.4%) | 22  (0.4%) | 16  (0.3%) | 65  (0.5%) | 32  (0.3%) | 33  (1.3%) | 14  (0.9%) | 33  (1.8%) | 126  (38.0%) |
| Throbbing or pressing | 313  (1.1%) | 52  (0.5%) | 35  (0.7%) | 17  (0.4%) | 70  (0.5%) | 33  (0.3%) | 37  (1.5%) | 20  (1.3%) | 33  (1.8%) | 138  (41.6%) |
| Location | 263  (1.0 %) | 45  (0.4%) | 24  (0.5%) | 21  (0.4%) | 52  (0.4%) | 15  (0.1) | 37  (1.5%) | 13  (0.9%) | 17  (0.9%) | 136  (41.0%) |
| Aggravation with physical activity | 539  (2.0%) | 144  (1.4%) | 58  (1.1%) | 86  (1.8%) | 184  (1.4%) | 99  (0.9%) | 85  (3.4%) | 31  (2.1%) | 44  (2.4%) | 136  (41.0%) |
| Nausea | 256  (0.9%) | 25  (0.2%) | 6  (0.1%) | 19  (0.4%) | 59  (0.4%) | 23  (0.2%) | 36  (1.4%) | 14  (0.9%) | 21  (1.1%) | 137  (41.3%) |
| Vomiting | 291  (1.1%) | 63  (0.6%) | 24  (0.5%) | 39  (0.8%) | 51  (0.4%) | 13  (0.1%) | 38  (1.5%) | 19  (1.3%) | 22  (1.2%) | 136  (41.0%) |
| Photophobia | 783  (2.9%) | 160  (1.6%) | 32  (0.6%) | 128  (2.6%) | 394  (2.9%) | 11  (0.1%) | 383  (15.3%) | 31  (2.1%) | 28  (1.5%) | 170  (51.2%) |
| Phonophobia | 470  (1.7%) | 87  (0.9%) | 16  (0.3%) | 71  (1.5%) | 181  (1.3%) | 22  (0.2%) | 159  (6.4%) | 16  (1.1%) | 25  (1.4%) | 161  (48.5%) |
|  |  |  |  |  |  |  |  |  |  |  |
| Missing in ≥1 diagnostic questions | 2430  (8.9%) | 763  (7.5%) | 385  (7.2%) | 378  (7.8%) | 1104  (8.2%) | 631  (5.8%) | 473  (18.9%) | 128  (8.6%) | 253  (13.7%) | 182  (54.8%) |
|  |  |  |  |  |  |  |  |  |  |  |
| Missing in all diagnostic questions | 130  (0.5%) | - | - | - | - | - | - | 4  (0.3%) | 7  (0.4%) | 119  (35.8%) |
